# Cross-Attention-Driven Propose-and-Select Generative Data Augmentation for Few-Shot Image Classification

**DOI:** 10.3390/s26144590

**Published:** 2026-07-20

**Authors:** Ying Liu, Liaomo Zheng, Shiyu Wang

**Affiliations:** 1 Shenyang Institute of Computing Technology, Chinese Academy of Sciences, Shenyang 110168, China; liuying221@sict.ac.cn (Y.L.); wangshiyu@sict.ac.cn (S.W.); 2 University of Chinese Academy of Sciences, Beijing 100049, China; 3 Shenyang CASNC Technology Co., Ltd., Shenyang 110168, China

**Keywords:** few-shot learning, data augmentation, diffusion models, candidate selection

## Abstract

Generative data augmentation based on diffusion models has emerged as a promising approach for few-shot image classification. Existing methods, such as DA-Fusion, typically follow a “generate-once, use-directly” paradigm, which often suffers from uncontrollable generation quality, unstable semantic consistency, insufficient global diversity, and high sample redundancy. To address these limitations, we propose a two-stage Propose-and-Select framework for controllable data augmentation. This framework curates high-quality synthetic data offline, ensuring that no additional training overhead is introduced to downstream models. For selector optimization, our method eliminates the need for additional human annotations by leveraging the zero-shot prior knowledge of a vision–language model (CLIP) to construct relative-quality pseudo-labels. Furthermore, we develop an adaptive-temperature listwise ranking distillation objective to transfer quality-aware supervision effectively. We also introduce a multi-objective consistency regularization strategy to stabilize training and improve convergence. Under a strictly controlled augmentation budget, where all methods are provided with the same number of synthetic samples, the proposed approach consistently outperforms existing diffusion-based augmentation baselines across both few-shot classification benchmarks, achieving an accuracy of 79.58% on PASCAL VOC and 80.74% on the fine-grained Oxford 102 Flowers dataset. These results demonstrate the effectiveness of the proposed generation-selection paradigm in improving the quality, diversity, and semantic relevance of synthetic samples, thereby enhancing downstream few-shot classification performance.

## 1. Introduction

Few-shot image classification aims to train visual models with strong generalization capability using only a limited number of labeled samples, and remains one of the fundamental challenges in computer vision research [1,2,3]. Traditional data augmentation methods increase the number of training samples through geometric or color transformations [4], such as flipping, rotation, and cropping [5,6]. However, since these techniques fail to introduce semantic-level diversity, their effectiveness is often limited in fine-grained recognition and few-shot scenarios [7,8]. To overcome the lack of semantic diversity, generative data augmentation has gradually emerged as an important research direction. Early generative augmentation approaches mainly relied on Generative Adversarial Networks (GANs) and Variational Autoencoders (VAEs) [9,10,11].

Nevertheless, under extremely limited training data, these conventional generative models exhibit inherent limitations and often struggle to synthesize high-fidelity images [12].

In recent years, text-to-image diffusion models have demonstrated remarkable image generation quality and powerful semantic modeling capabilities [13,14,15], providing new opportunities for few-shot learning. Existing diffusion-based data augmentation methods, such as DA-Fusion [16], have adapted these models to novel domains and fine-grained concepts. Despite these advances, existing diffusion-based augmentation methods still suffer from several limitations [17]. First, the generation process is largely uncontrollable. Consequently, generated samples may exhibit image distortions, semantic drift, or other artifacts [18,19]. Second, there is no explicit quality evaluation mechanism. Existing methods generally lack effective scoring and filtering strategies for generated samples, making it impossible to distinguish high-quality samples from low-quality ones. As a result, noisy synthetic samples may directly degrade downstream model performance [20]. Third, global diversity is often insufficient. Repeated augmentation tends to produce homogeneous samples within the same category, increasing the risk of overfitting and mode collapse [21]. Fourth, current training strategies fail to model relative relationships among candidate samples. As a result, the selection process is often unstable and unreliable, while important constraints such as diversity preservation and permutation consistency are rarely considered [22]. To address the above challenges, this paper proposes a controllable generative augmentation framework driven by a Propose-and-Select paradigm. Coupled with a lightweight cross-attention selector and a diversity memory bank, our framework establishes a rigorous multidimensional scoring mechanism to dynamically penalize redundant noise and ensure high-fidelity data synthesis.

Recent advances in diffusion models have significantly promoted controllable image generation and data augmentation for low-data scenarios. Methods based on Stable Diffusion, ControlNet, and instruction-guided editing have demonstrated remarkable capability in generating diverse synthetic samples while preserving semantic consistency. Meanwhile, recent few-shot learning approaches have increasingly leveraged vision–language models such as CLIP through prompt learning, adapter tuning, and prototype adaptation to improve generalization under limited supervision. Despite these advances, existing diffusion-based augmentation methods generally rely on one-shot generation or heuristic filtering strategies, lacking an explicit mechanism for jointly optimizing semantic quality and dataset-level diversity. This limitation motivates the proposed Propose–Select framework [23,24,25].

The remainder of this paper is organized as follows. Section 2 reviews related work on diffusion-based data augmentation, few-shot learning, and sample selection methods. Section 3 presents the proposed Propose–Select framework and its implementation details. Section 4 describes the experimental settings and reports both ablation studies and comparative evaluations. Finally, Section 5 concludes the paper and discusses future research directions.

## 2. Related Work

The objective of this study is to address the challenges of few-shot image classification by generating high-quality synthetic data. The proposed approach relies on the deep integration of generative models, data augmentation strategies, and sample refinement mechanisms.

### 2.1. Diffusion Models and Generative Data Augmentation

In recent years, diffusion models have emerged as the dominant paradigm for generative data augmentation due to their remarkable image fidelity and strong cross-modal semantic alignment capabilities. Techniques such as SDEdit [26], Textual Inversion, ControlNet, and instruction-guided image editing have substantially improved the controllability of synthetic image generation. Building upon these advances, DA Fusion and other methods further utilize diffusion models to expand the training datasets for few-shot learning, leading to improved downstream classification performance [27]. Meanwhile, recent few-shot learning approaches have increasingly incorporated vision–language models through prompt learning, adapter tuning, and prototype adaptation to enhance generalization under limited supervision. Nevertheless, most existing diffusion-based augmentation frameworks still follow a single-candidate direct generation paradigm, in which generated samples are directly incorporated into the training set or filtered using heuristic strategies without explicitly modeling sample quality and dataset-level diversity. Consequently, semantic drift, low-quality samples, and feature redundancy remain challenging issues.

To overcome these limitations, this work departs from conventional generation-only strategies by introducing a candidate selection mechanism. By integrating the generative capability of diffusion models with a controllable selection framework, a controllable generative augmentation paradigm is established to improve sample quality, diversity, and reliability.

### 2.2. Few-Shot Learning and Data Augmentation

The primary objective of few-shot learning is to train models with strong generalization capability using only a limited number of labeled samples. Existing approaches mainly include meta-learning, metric learning, and model fine-tuning. Within these frameworks, data augmentation serves as a fundamental technique for mitigating overfitting and improving model generalization.

In few-shot learning scenarios, a significant imbalance often exists between the large number of model parameters and the limited amount of available training data. This imbalance frequently results in severe overfitting and degraded generalization performance. Traditional data augmentation methods mainly rely on geometric transformations, such as random cropping and affine transformations, as well as pixel-level perturbations, including color jittering and noise injection. Although these strategies can increase the number of training samples at relatively low computational cost, they essentially perform only minor perturbations around the low-dimensional manifold of the original data distribution. Since they cannot modify high-level semantic information, their effectiveness remains limited for challenging tasks such as fine-grained recognition and novel-class generalization.

To overcome the limitations of conventional augmentation, generative data augmentation has gradually become an important research direction. By synthesizing novel samples containing previously unseen semantic variations, generative approaches aim to fill gaps in the underlying data distribution. However, under extreme few-shot conditions, traditional generative models often suffer from training instability and mode collapse, making it difficult to generate images that simultaneously exhibit high fidelity and sufficient diversity.

Benefiting from rich zero-shot priors, diffusion models have recently emerged as a powerful solution to overcome these traditional bottlenecks, providing semantically meaningful and visually diverse image variants from only a few reference samples. Although diffusion-based generative augmentation has become a major research focus, quality control and diversity preservation remain critical challenges. Our work specifically addresses the challenges of quality control and diversity by introducing a candidate selection mechanism and a diversity memory bank, thereby establishing a controllable and high-quality augmentation pipeline for diffusion-generated data. The proposed framework provides a new perspective for enhancing few-shot learning through reliable generative data augmentation.

### 2.3. Sample Selection and Learning-to-Rank

Under the multi-candidate generation paradigm, sample selection [28,29] becomes a critical component for refining the effectiveness of data augmentation. Its primary objective is to identify a subset of samples with higher quality and greater diversity from a pool of generated candidates.

Learning-to-rank provides an important theoretical foundation for sample evaluation and selection. Existing ranking approaches can generally be categorized into pointwise, pairwise, and listwise methods. Traditional pointwise approaches treat each candidate image as an independent instance and predict an absolute quality score. However, because they fail to explicitly model the relative differences among candidates within the same candidate set, their discriminative capability often deteriorates when applied to diffusion-generated images whose quality distributions are highly concentrated. Pairwise methods introduce binary comparisons between samples, but they lack a global view of the quality distribution across the entire candidate set and typically incur higher computational complexity. In contrast, listwise ranking methods treat the entire candidate set as a single training instance and directly model the relative relationships among candidates, thereby enabling more accurate and robust ranking performance.

CLIP learns a shared embedding space for visual and textual representations through contrastive learning on large-scale image-text pairs. Owing to its strong cross-modal semantic alignment capability, CLIP has been widely adopted for image-text and image-image similarity measurement, providing an effective tool for sample quality assessment. In extreme few-shot augmentation scenarios, obtaining additional human annotations for generated-image quality is often prohibitively expensive and impractical. The strong zero-shot generalization capability of CLIP makes it a suitable quality prior estimator without requiring task-specific supervision.

In the proposed Select Phase, high-dimensional semantic features extracted by CLIP are utilized to construct explicit multi-dimensional quality pseudo-labels. Specifically, the selector computes cosine similarities between candidate images and three key references: the target-class text descriptions, the original source images, and the global memory bank. These similarity measurements provide reliable quality supervision signals without introducing any additional trainable parameters. The resulting pseudo-labels are subsequently used to guide the training of the Cross-Attention Selector.

In this work, the screening of diffusion-generated candidates is formulated as a listwise ranking distillation. Semantic representations extracted by CLIP are employed to construct quality-aware ranking signals, while a lightweight Cross-Attention Selector is designed to model interactions among candidates and perform accurate ranking and selection. Furthermore, a composite loss function is introduced to optimize the selector training process, resulting in improved selection reliability, semantic consistency, and dataset diversity.

### 2.4. Comparison with Existing Sample Filtering Paradigms

While sample filtering methods (e.g., QDE [20]) have taken the first step toward quality-aware augmentation, their effectiveness is fundamentally limited by their reliance on static or heuristic-based filtering criteria. In contrast, our proposed Propose-and-Select framework introduces three architectural advancements that achieve a higher level of controllable augmentation:

- Cross-Attention Set Interaction vs. Static Scoring: Unlike QDE, which typically evaluates samples in isolation, our cross-attention set interaction module explicitly models the joint semantic relationship between candidate images and target class representations. This allows for fine-grained semantic alignment that heuristic metrics cannot capture.
- Adaptive-Temperature Listwise Distillation vs. Pointwise Optimization: Existing methods usually optimize filtering criteria on a sample-by-sample basis (pointwise). We innovate by introducing adaptive-temperature listwise ranking distillation, which optimizes the relative quality order of the entire candidate pool. This preserves the subtle rank-order relationships, leading to more stable and robust supervisor signals for the selector.
- Class-Level Diversity Memory Bank vs. Global Filtering: Most previous approaches lack mechanisms to prevent redundancy across generated samples. Our class-level diversity memory bank maintains a dynamic history of the generated distribution, providing an explicit penalty against structural and semantic repetition. This goes beyond the scope of QDE, which primarily focuses on individual image quality, to effectively mitigate the long-standing mode collapse issue in diffusion-based few-shot augmentation.

## 3. Method

### 3.1. Overall Framework

**Figure 1:**
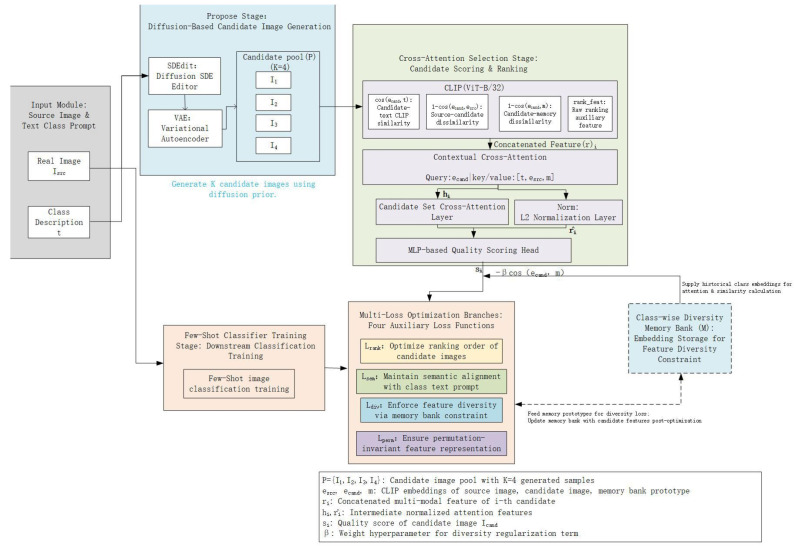
System Framework.

A two-stage Propose-and-Select controllable generative augmentation framework is proposed in this work. The overall architecture of the proposed framework is illustrated in Figure 1.

The proposed framework consists of three core stages:

Propose Stage: For each real image $I_{\mathrm{src}}$, a diffusion model (Stable Diffusion v1.5) is employed to generate *K* augmented candidate images, $\left\{I_{1},I_{2},\ldots ,I_{K}\right\}$, thereby constructing a diverse candidate pool for subsequent selection.

Select Stage: The generated candidate images are fed into the proposed Cross-Attention Selector. By jointly modeling explicit quality features, contextual information, and inter-candidate interactions, the selector evaluates and ranks all candidates, ultimately selecting the most suitable augmented image $I_{\mathrm{best}}$ with high semantic fidelity and enhanced diversity.

Model Training Stage: The selected augmented images are combined with the original real images to form the final training set. A downstream classifier is then trained on this mixed dataset, enabling improved generalization performance in few-shot classification scenarios.

### 3.2. Propose Stage

To address the instability of generated image quality caused by the heavy reliance on the stochasticity of diffusion models in existing data augmentation methods (e.g., DA-Fusion), the proposed framework adopts the SDEdit strategy in the Propose stage to perform image editing and augmentation.

The key idea of this stage is to construct a candidate pool containing multiple augmented samples for each real image rather than generating a single augmented image. Specifically, given a source image, a candidate set $P=\{I_{1},I_{2},\ldots ,I_{K}\}$ is generated, providing a rich search space for the subsequent selection stage. In this work, the pre-trained Stable Diffusion v1.5 model is employed as the image generation engine, while SDEdit is utilized to perform controlled image augmentation. The generation process is configured as follows:

Latent Space Transformation: The source image $I_{\mathrm{src}}$ is first encoded into the latent space through a Variational Autoencoder (VAE), enabling subsequent diffusion-based image editing operations.

Noise Injection and Denoising: The noise strength is set to 0.5 to balance image fidelity and diversity. Under this configuration, approximately 50% of the structural information from the source image is preserved, while sufficient semantic variation can be introduced during the denoising process.

Guidance Scale: The classifier-free guidance (CFG) scale is set to 7.5 to encourage semantic consistency between the generated images and their corresponding text prompts.

Number of Candidates: The candidate pool size is fixed at K=4, achieving a trade-off between generation quality and computational cost. The effectiveness of this setting is further validated through sensitivity analysis.

With the above configuration, four semantically plausible yet structurally diverse augmented images are generated for each source image. These candidates serve as the foundation for the subsequent Select stage, which aims to identify high-quality and diverse augmentation samples.

### 3.3. Select Stage: Cross-Attention Candidate Quality Selector

The Select stage constitutes the core component of the proposed augmentation framework, aiming to identify the most suitable sample from the candidate images generated in the Propose stage. An ideal augmented sample should not only exhibit high visual quality and semantic consistency but also provide sufficient diversity to improve the generalization capability of the downstream classifier.

However, directly employing external assessment networks to evaluate candidate images is often impractical in few-shot scenarios. Such approaches introduce substantial computational overhead and are prone to overfitting due to the limited availability of training data.

To address these challenges, we propose a Cross-Attention Candidate Quality Selector, a lightweight ranking network that leverages the zero-shot prior knowledge of CLIP to extract explicit multi-dimensional quality features. By integrating these handcrafted quality cues with a cross-attention mechanism, the selector can effectively model both the intrinsic quality of individual candidates and the relationships among multiple candidates within the same candidate pool. Compared with conventional evaluation networks, the proposed selector requires significantly fewer learnable parameters, enabling more reliable quality assessment under limited-data conditions. Specifically, the proposed Cross-Attention Candidate Quality Selector consists of the following four key components.

#### 3.3.1. Explicit Quality Features

To provide informative quality cues for candidate evaluation, we leverage the CLIP (ViT-B/32) model to extract vision–language features and construct three complementary quality signals. Specifically, let $e_{cand}\in R^{d}$ denote the feature embedding of the *i*-th candidate image, $t\in R^{d}$ denote the category text embedding, $e_{src}\in R^{d}$ denote the source image embedding, and $m\in R^{d}$ denote the average embedding stored in the diversity memory bank, where d=512 is the unified cross-modal feature dimension of CLIP. Based on these representations, three key quality signals are computed to characterize the semantic relevance, diversity, and redundancy of each candidate image.

Semantic Alignment:Semantic alignment measures the consistency between a candidate image and its corresponding category description. It is computed as the cosine similarity between the candidate image embedding and the category text embedding. A higher value indicates stronger semantic consistency with the target category.

Source-image Diversity: To quantify the degree of variation introduced by augmentation, the diversity between a candidate image and the source image is measured as $1-\cos(e_{cand},e_{\mathrm{src}})$. A lower value indicates that the generated image remains highly similar to the source image and therefore provides limited diversity.

Memory Redundancy:Memory redundancy quantifies the feature distance between a candidate image and the historical distribution stored in the diversity memory bank. It is computed as $1-\cos(e_{cand},m)$. A lower value indicates a smaller difference between the candidate image and the images within the memory bank, reflecting a higher degree of redundancy.

By concatenating three types of signals with ranking features, an explicit quality feature vector is constructed:

(1) $$f_{i}=\left[\cos\left(e_{\mathrm{cand}},t\right),1-\cos\left(e_{\mathrm{cand}},e_{\mathrm{src}}\right),1-\cos\left(e_{\mathrm{cand}},m\right),r_{\mathrm{feat}}\right]$$

#### 3.3.2. Context Cross-Attention

To enable candidate images to fully leverage contextual information (including category text, the original image, and the diversity repository) for self-evaluation, this paper employs a contextual cross-attention mechanism.

This module treats the category text, the source image, and the diversity repository as three complementary reference sources to construct a global representation space for candidate images. Specifically, the embedding of a candidate image is used as the query vector, while the features derived from the three reference sources are concatenated to form the key and value inputs of the attention layer. This allows the model to learn context-aware feature representations through cross-attention:

(2) $$h_{i}=\mathrm{CrossAttn}\left(e_{cand},\;\left[t,e_{\mathrm{src}},m\right]\right)$$

By incorporating attention mechanisms, this module enables candidate images to better capture semantic consistency, diversity, and other quality-related aspects. The resulting hidden states encode high-order contextual interactions, providing a strong foundation for subsequent scoring.

#### 3.3.3. Cross-Attention Mechanism

Compared to conventional independent single-image scoring networks, our cross-attention-driven selector offers three critical advantages:

- Dynamic Context-Aware Evaluation: Each candidate feature $e_{cand}$ is contextualized by all other candidates within the pool. This enables the selector to detect feature-level redundancies that static pointwise networks inherently miss. For instance, if $e_{cand}$ is highly similar to another candidate in the pool, the cross-attention weights dynamically redistribute, naturally penalizing redundant samples.
- Global Feature Alignment: The cross-attention mechanism projects candidate features into a shared relational space. Here, the relative contribution of each sample towards the target text embedding *t* is explicitly computed. This allows the model to perform robust ranking based on relative semantic alignment rather than relying on rigid, absolute pointwise thresholds.
- Implicit Diversity Enforcement: Independent scoring networks are fundamentally blind to the overall distribution of the candidate set. By explicitly modeling the dense interaction matrix among all candidates, our selector promotes the selection of diverse samples that collectively cover the semantic manifold, effectively preventing “structural homogenization”.

#### 3.3.4. Set Interaction Layer

To model the relative relationships among candidate images and achieve more accurate quality ranking, this paper further designs a candidate set interaction layer within the selector. This layer employs a self-attention mechanism to enable interactions among all candidate images in a fully connected manner. Specifically, it takes the contextual feature of each candidate image as input and applies the self-attention layer to learn inter-candidate relationships, thereby producing more robust feature representations.

(3) $${\hat{h}}_{i}=\mathrm{SelfAttn}\left(h_{i},\left\{h_{1},\ldots ,h_{K}\right\}\right)$$

Instead of scoring each candidate image independently, this module dynamically compares the features of each candidate with those of all other candidates, thereby incorporating global relational information during feature aggregation. This feature fusion strategy enables the model to better distinguish between high- and low-quality samples, thereby improving ranking accuracy.

#### 3.3.5. Quality Score Head

As the final step in the selector evaluation process, the quality scoring head aims to effectively fuse high-level candidate interaction features with low-level explicit quality priors to produce the final relative quality score. It concatenates the features from the contextual cross-attention with the explicit quality features and uses an MLP to output the final quality score for each candidate image:

(4) $$s_{i}=\mathrm{MLP}\left(\left[{\hat{h}}_{i},{\tilde{f}}_{i}\right]\right)$$

${\tilde{f}}_{i}$ denotes the normalized explicit quality feature vector. This combined input enables the model to maintain a macro-level understanding of the relative quality across the candidate set while preserving the original cosine similarity signal during forward propagation in the deep network. During inference, the selector ultimately chooses the candidate image with the highest score as $I_{\mathrm{best}}$.

### 3.4. Diversity Memory Bank

To mitigate sequence information redundancy caused by repeated augmentation and to preserve the global diversity of the augmented dataset, this study introduces a diversity memory bank for each target class. The memory bank continuously records and tracks the embedding features of previously selected augmented images. Its operational mechanism is described as follows:

- Memory Bank Initialization: At the beginning of the augmentation process, an initially empty memory bank is allocated for each class. This memory bank is used to persistently store CLIP feature embeddings of augmented images selected by the selector.
- Memory Bank Update: After the selection of $I_{\mathrm{best}}^{\left(t\right)}$ in the Select stage of the *t*-th round, its feature embedding $e_{\mathrm{best}}^{\left(t\right)}$ is immediately extracted and stored in the corresponding class-specific memory bank.
- Inference-Time Re-ranking: During the generation of augmented images, the similarity between each candidate image and the feature representations stored in the corresponding memory bank is computed. A diversity-aware penalty is then applied to the quality score to discourage the selection of samples that are overly similar to previously selected augmented images.
  (5) $${\hat{s}}_{i}=s_{i}-\beta \cdot \cos\left(e_{cand},M\right)$$
  where *M* denotes the average embedding of previously selected images stored in the memory bank, and β is the diversity penalty coefficient. The proposed diversity memory bank prevents the repeated selection of highly similar samples during sequential generation. Consequently, this penalty strategy preserves the global diversity of the augmented dataset. Consequently, it effectively alleviates the issue of mode collapse that commonly arises in few-shot learning scenarios.

The sequential execution flow of the diversity-aware selection is summarized in Algorithm 1. The re-ranking process dynamically adjusts the selector’s scores by subtracting the similarity component $\beta \cdot \cos(e_{cand},M)$. This ensures that candidates semantically overlapping with the already-augmented set are explicitly penalized, forcing the model to explore less-represented regions of the semantic manifold.

The memory update logic follows the chosen MEM strategy (FIFO, Reservoir, or Momentum) as discussed in Section 4.4.3, ensuring that the bank M remains a robust representation of the historical selection distribution. By executing this update in each sequential round, the framework avoids the mode collapse issue inherent in unconstrained generative augmentation.

### 3.5. Composite Loss Function

To ensure stable training of the selector, this study designs a composite loss function consisting of four components, jointly optimizing ranking accuracy, semantic consistency, diversity, and permutation equivariance. Existing ranking screening methods typically optimize for absolute image quality, which often inadvertently compromises semantic alignment or global diversity. To address this limitation and achieve what existing methods fail to do simultaneously, we formulate a composite training objective *L* that enforces four complementary constraints:

(6) $$L=\lambda_{\mathrm{rank}}L_{\mathrm{rank}}+\lambda_{\mathrm{sem}}L_{\mathrm{sem}}+\lambda_{\mathrm{div}}L_{\mathrm{div}}+\lambda_{\mathrm{perm}}L_{\mathrm{perm}}$$

where $\lambda_{rank}$, $\lambda_{sem}$, $\lambda_{div}$, and $\lambda_{perm}$ denote the weighting coefficients for each loss term.
**Algorithm 1 **Diversity-Aware Sequential Selection and Memory Update**Input**: Candidate pool $C=\{c_{1},...,c_{N}\}$, initial scores $S=\{s_{1},...,s_{N}\}$, embeddings $E=\{e_{1},...,e_{N}\}$, penalty weight β, selection size *K*, memory strategy STRATEGY.**Initialize**: Augmented set A←∅, Memory Bank M  1:**for **k=1 to *K* **do**  2:   **for** each remaining candidate $c_{i}\in C$ **do**  3:     // Calculate semantic overlap with Memory Bank  4:     $Penalty_{i}\leftarrow \cos\left(e_{cand},M\right)$  5:     *// Re-rank via explicit diversity penalty*  6:     ${\hat{s}}_{i}\leftarrow s_{i}-\beta \cdot Penalty_{i}$  7:   **end for**  8:   *// Select the optimal candidate for current round*  9:   $c^{*}\leftarrow \arg\max_{c_{i}\in C}{\hat{s}}_{i}$10:   $A\leftarrow A\cup \{c^{*}\}$11:   $C\leftarrow C\setminus \{c^{*}\}$12:   *// Dynamic Memory Update based on chosen strategy*13:   **if** STRATEGY == FIFO **then**14:     $M\leftarrow \mathrm{UpdateQueue}(M,e_{c^{*}})$15:**   else if **STRATEGY == Reservoir **then**
16:     $M\leftarrow \mathrm{ReservoirSample}(M,e_{c^{*}},k)$17.   **else if** STRATEGY == Momentum **then**
18:     $M\leftarrow m\cdot M+\left(1-m\right)\cdot e_{c^{*}}$19:   **end if**20:**end for**21:**return** Final diverse augmented set A

This multi-objective optimization framework stabilizes the selector training by ensuring that each regularization term addresses a specific vulnerability of the candidate screening process:

- Listwise Ranking Loss ($L_{rank}$): This serves as the primary objective, leveraging quality signals derived from CLIP to distill supervision for candidate ranking. While effective for relative ordering, relying solely on this term can lead to semantic drift and redundancy within the generated set.
- Semantic Consistency Loss ($L_{sem}$): To complement the relative ranking, this term acts as a soft regularization that encourages the selector’s ranking distribution to align with absolute semantic supervision signals. It ensures the semantic reliability of the selection process by preventing the model from favoring candidates that are visually impressive but semantically misaligned.
- Diversity Regularization Loss ($L_{div}$): Existing screening methods often suffer from mode collapse by repeatedly selecting visually similar high-quality samples. To combat this, $L_{div}$ explicitly penalizes candidates that are highly similar to previously stored memory representations and introduces an entropy term to discourage overly peaked score distributions. It guarantees the dataset-level diversity that $L_{rank}$ and $L_{sem}$ inherently miss.
- Permutation Equivariance Loss ($L_{perm}$): Since the candidate images are generated in parallel, their input order to the cross-attention selector is entirely arbitrary. This regularization term enforces that the predicted quality scores remain strictly consistent regardless of how the input candidates are shuffled. By penalizing variations in the output distribution caused by positional changes, it ensures the theoretical soundness and stability of the cross-attention mechanism, preventing it from overfitting to positional biases.

#### 3.5.1. Listwise Ranking Loss

This loss serves as the primary objective for training the selector, leveraging quality signals derived from CLIP to distill supervision for candidate ranking. Specifically, a teacher signal is first constructed as follows:

(7) $$r_{i}=\cos\left(e_{\mathrm{cand}},t\right)-\alpha \cos\left(e_{\mathrm{cand}},e_{\mathrm{src}}\right)$$

where α is a weighting coefficient that controls the strength of the diversity penalty. Since the value range of CLIP-based scores is relatively limited in few-shot scenarios, using a fixed temperature may lead to an overly smooth teacher distribution and consequently cause gradient vanishing issues. Therefore, this study adopts an adaptive temperature mechanism:

(8) $$\tau_{\mathrm{eff}}=\max\left(\frac{\mathrm{range}(r)}{10},\tau_{\min}\right)$$

where range(r) denotes the range of the teacher signal *r*, and $\tau_{\min}$ is the minimum temperature threshold, set to 0.003 in this study. The adaptive temperature dynamically adjusts according to the distribution of the teacher signal, sharpening the teacher distribution and thereby improving the effectiveness of knowledge distillation. The listwise ranking distillation is then defined by measuring the discrepancy between the student distribution (i.e., the score distribution produced by the selector) and the teacher distribution using KL divergence:

(9) $$L_{\mathrm{rank}}=D_{\mathrm{KL}}\left(p\;∥\;q_{\mathrm{smooth}}\right)$$

where $q_{\mathrm{smooth}}$ denotes the student distribution, and *p* represents the sharpened teacher distribution.

#### 3.5.2. Semantic Consistency Loss

To encourage the selector to prefer candidate images with high semantic alignment, a semantic consistency loss is introduced as follows:

(10) $$L_{\mathrm{sem}}=-\sum_{i}p_{i}\cdot r_{i}$$

where $p_{i}$ denotes the probability distribution over candidate images produced by the selector, and $r_{i}$ represents the corresponding teacher signal. This loss acts as a soft regularization term that encourages the selector’s ranking distribution to align with semantic supervision signals, thereby improving the semantic reliability of the selection process.

#### 3.5.3. Diversity Regularization Loss

To ensure diversity among the selected results, a diversity regularization loss is introduced as follows:

(11) $$L_{\mathrm{div}}=\sum_{i}p_{i}\cdot m_{i}-\lambda_{\mathrm{ent}}\;H\left(p\right)$$

where $m_{i}$ denotes the similarity between the candidate image and the diversity memory bank, H(p) represents the entropy of the score distribution, and $\lambda_{\mathrm{ent}}$ is the entropy regularization coefficient, set to 0.01. This loss penalizes candidates that are highly similar to previously stored memory representations, while the entropy term discourages overly peaked score distributions. Together, these two components promote diverse selection results.

#### 3.5.4. Permutation Consistency Loss

To enforce permutation invariance of the selector with respect to candidate inputs, we introduce a permutation consistency loss. The input candidate set is randomly shuffled to obtain a perturbed version, and the selector produces a corresponding distribution $p^{\prime }$. The discrepancy between *p* and $p^{\prime }$ is measured using bidirectional KL divergence:

(12) $$L_{\mathrm{perm}}=D_{\mathrm{KL}}\left(p\;∥\;p^{\prime }\right)+D_{\mathrm{KL}}\left(p^{\prime }\;∥\;p\right)$$

This loss improves the robustness and generalization ability of the selector by enforcing permutation invariance.

## 4. Results

Through the aforementioned Propose-and-Select framework and the composite loss optimization, our method theoretically guarantees the generation of high-quality, semantically aligned, and visually diverse augmented candidates. To empirically validate the effectiveness of these theoretical designs, Section 4 will present comprehensive evaluations. We aim to demonstrate how this explicit quality control translates into tangible performance gains for downstream few-shot classification models across different domains.

### 4.1. Experimental Settings

#### 4.1.1. Datasets

The experiments utilized two types of datasets:the PASCAL VOC 2012 dataset and the Oxford 102 Flowers dataset.

(1) PASCAL VOC 2012 Dataset: It consists of 11,400 images across 20 categories of natural scenes.
(2) Oxford 102 Flowers dataset: A dataset for fine-grained classification of flower species, containing 102 different categories.

#### 4.1.2. Baseline Methods

We compare the proposed method with several representative data augmentation and few-shot learning baselines, including both traditional augmentation strategies and recent generative or selection-based approaches. The baseline methods are listed as follows:

- No-Aug: No data augmentation is applied; the classifier is trained directly using the original 4-shot labeled samples;
- RandAugment: Automatic image enhancement external baseline;
- AugMix: Robust image enhancement with external baseline;
- TrivialAugmentWide: Automatic enhancement of external baseline without parameter tuning;
- DA-Fusion: A state-of-the-art diffusion-based data augmentation method that adopts a single-candidate generation strategy, incorporating text inversion and SDEdit for unconstrained generation without any selection process;
- Propose–Select: Two-stage controllable generation enhancement framework.

#### 4.1.3. Experimental Setup

- Hardware Configuration: Intel i7-12700K CPU, NVIDIA RTX 3090 GPU (24 GB VRAM), and 32 GB DDR4 RAM.
- Software Environment: Python 3.9-PyTorch 2.0-Ultralytics 8.1-CUDA 11.7;
- Training Strategy: To strictly prevent data leakage and decouple the generation evaluation from the classification task, our training pipeline is divided into two independent phases:
  Phase 1: Offline Selector Optimization
  To ensure strictly zero data leakage, the Cross-Attention Selector is pre-trained purely on a large-scale independent external dataset (e.g., MS-COCO 2017) to learn universal quality-aware representations. It is optimized using the SGD optimizer with a learning rate of 0.01 for 50 epochs, guided by the proposed composite loss. Once optimized, all parameters of the selector are strictly frozen for downstream tasks.
  Phase 2: Downstream Classifier Training
  For the actual few-shot classification on target datasets (i.e., PASCAL VOC 2012 and Oxford 102 Flowers), the frozen selector is utilized solely as an offline inference module to curate generated candidates. The downstream classification model is trained for 50 epochs using the SGD optimizer and standard cross-entropy loss. Because the selector remains frozen, this process introduces negligible computational overhead to the main classification pipeline.

### 4.2. Evaluation Metrics

The evaluation metrics in this study include the following four indicators. The average best validation accuracy (Val Acc) is used to assess classification performance, while semantic alignment (CLIP Sim), perceptual diversity (LPIPS), and CLIP Feature Diversity are employed to evaluate the quality of the generated data [30].

- Val Acc: To evaluate the model’s generalization performance on few-shot classification tasks, validation accuracy is adopted as the primary metric. It is defined as the proportion of correctly classified samples on the validation set. Considering the inherent training instability of deep models, we further report the best validation accuracy, which is computed as the highest average validation accuracy achieved over the entire training process;
- CLIP Sim: To measure the semantic consistency between the generated augmented images and the target classes, CLIP Similarity (CLIP Sim) is adopted as the semantic alignment metric. This metric leverages a pretrained vision–language model (CLIP) to extract feature embeddings of both images and textual prompts, and computes their semantic matching degree via cosine similarity. A higher CLIP Sim score indicates a stronger alignment between the generated images and the target class in the shared semantic space;
- LPIPS: It is a metric used to measure the perceptual similarity between two images, where a lower value indicates higher similarity;
- CLIP Feature Diversity: To quantitatively evaluate the global CLIP feature diversity of augmented samples, we define the CLIP Feature Diversity metric. Given a set of augmented images $I=\{i_{1},i_{2},\ldots ,i_{N}\}$ for a specific category, we extract their deep feature representations $E=\{e_{1},e_{2},\ldots ,e_{N}\}$ using a pre-trained CLIP vision encoder. The CLIP Feature Diversity is defined as the average pairwise cosine distance among all these CLIP embeddings:
  (13) $$CLIPFeatureDiversity=\frac{1}{N(N-1)}\sum_{j=1}^{N}\sum_{k\neq j}\left(1-\cos\left(e_{j},e_{k}\right)\right)$$
  where cos(·,·) denotes the cosine similarity function. A higher CLIP Feature Diversity indicates greater global feature diversity among the augmented samples, reflecting the capability of an augmentation strategy to generate semantically diverse data while reducing redundant sample distribution.

### 4.3. Comparative Results

A comparison is conducted between the proposed augmentation model, No-aug, and DA-Fusion in terms of classification performance, and the quality of the generated data produced by each method is further evaluated. All perceptual metrics (LPIPS, CLIP Sim, CLIP Feature Diversity) are reported with four decimal places forconsistent precision across all tables.

#### 4.3.1. Results on the PASCAL VOC 2012 Dataset

Having established the foundational evaluation protocols and implementation details, we investigate the direct impact of our framework on downstream classification tasks using the PASCAL VOC 2012 benchmark. All reported results are averaged over five independent runs to ensure statistical significance. The classification performance is summarized in Table 1.

- No-aug Baseline: Under the extreme few-shot setting, the classification accuracy is only 72.91%±0.81%. The relatively high standard deviation indicates that the model is highly susceptible to the stochastic nature of few-shot data splits, leading to severe overfitting.
- Traditional Augmentation Baselines (RandAugment, AugMix, TrivialAugmentWide): Applying conventional pixel-level data augmentation strategies yields marginal performance gains, achieving accuracies of 73.02% ± 0.49%, 73.91% ± 0.52%, and 73.02% ± 0.43%, respectively. Although these methods slightly reduce the standard deviation compared to the No-aug setting (indicating marginally improved stability), their overall accuracy remains bottlenecked. This highlights a fundamental limitation: traditional geometric and color transformations fail to introduce novel semantic information or structural diversity, which are critically needed to overcome severe data scarcity in the extreme few-shot regime.
- DA-Fusion Baseline: The introduction of standard diffusion-based augmentation improves the accuracy to 77.65%±0.64%. However, this paradigm lacks an effective quality-aware selection mechanism, resulting in a moderate variance across different runs due to the presence of semantically misaligned and low-diversity noise.
- Propose–Select (Ours): By incorporating the proposed cross-attention selector and composite supervision mechanism, our framework achieves an accuracy of 79.58%±0.26%. Notably, our method not only outperforms the DA-Fusion baseline by 1.93 percentage points but also exhibits the smallest standard deviation among all methods. This significantly reduced variance provides empirical evidence that our framework successfully stabilizes the augmentation process against the inherent stochasticity of diffusion-based generation.

To further analyze the impact of augmented data on classification performance, we quantitatively evaluate the quality of generated samples from three perspectives: CLIP Sim, LPIPS, and CLIP Feature Diversity. The results are reported in Table 2.

- CLIP Sim: The proposed Propose–Select framework achieves the highest CLIP Sim score of 0.2789±0.0216 among all compared methods, surpassing RandAugment (0.2651±0.0179), AugMix (0.2688±0.0177), TrivialAugmentWide (0.2653±0.0178), and DA-Fusion (0.2455±0.0274). This improvement demonstrates that the cross-attention selector effectively leverages CLIP-based semantic priors to evaluate and filter generated candidates. Compared with conventional augmentation strategies, which mainly rely on hand-crafted transformations without explicit semantic guidance, and diffusion-based generation methods that may introduce semantic drift, Propose–Select better preserves the category-level semantic consistency of augmented samples.
- LPIPS: In terms of perceptual diversity measured by LPIPS, the proposed method achieves 0.4424±0.0286, which is higher than DA-Fusion (0.4102±0.0321) and AugMix (0.4666±0.0412) while maintaining superior semantic alignment. Although RandAugment and TrivialAugmentWide obtain relatively higher LPIPS values of 0.5969±0.0466 and 0.5797±0.0857 respectively, these methods introduce diversity through predefined geometric and photometric transformations rather than semantic-aware generation. In contrast, Propose–Select achieves an optimal balance between perceptual variation and semantic fidelity by selecting high-quality diffusion-generated samples from diverse candidate sets.
- CLIP Feature Diversity: The proposed method achieves a CLIP Feature Diversity of 0.0828±0.0245, which is significantly lower than DA-Fusion (0.1856±0.0470) and conventional augmentation baselines (which consistently exceed 0.23). It is crucial to note that a higher diversity score does not necessarily equate to a superior augmentation strategy. Conventional methods often yield high feature variance through aggressive, unconstrained geometric or photometric perturbations, which severely distort the original semantic structure and ultimately degrade downstream classification performance. In contrast, our approach strikes an optimal balance. By penalizing samples that are overly similar to the historical distribution, the introduced diversity memory bank effectively reduces feature redundancy and alleviates the *mode collapse* issue during sequential generation. Consequently, our method maintains a reasonable and controlled feature diversity while strictly preserving semantic fidelity (achieving the highest CLIP Sim) and maximizing downstream accuracy, thereby ensuring the global robustness of the augmented dataset.

#### 4.3.2. Results on the Oxford 102 Flowers

To further validate the generalization capability of our Propose–Select framework, we extended our evaluation to the Oxford 102 Flowers dataset, which presents unique challenges due to high intra-class variance and fine-grained structural details. The classification performance and data quality evaluation results are summarized in Table 3 and Table 4, respectively.

- Failure of Traditional Augmentation on Fine-grained Data: Unlike standard object recognition tasks, applying traditional pixel-level augmentations (e.g., RandAugment, AugMix) on the Oxford 102 Flowers dataset leads to a notable performance degradation (dropping from the No-aug baseline of 72.50% to 69.34%∼70.57%). This negative transfer occurs because aggressive geometric distortions and color jittering blindly destroy the delicate fine-grained features (such as petal textures and specific color distributions) that are critical for floral classification.
- Superiority and Stability of Propose–Select (Ours): Our framework consistently outperforms all existing baselines, achieving an average best validation accuracy of 80.74%±0.32%, which is 2.24 percentage points higher than the unconstrained DA-Fusion baseline. More importantly, the significantly smaller standard deviation (0.32% vs. 0.49%) confirms that our cross-attention selector effectively stabilizes the training process. By strictly anchoring the semantic identity, our method successfully preserves the fine-grained nature of floral features while expanding structural diversity, effectively overcoming the inherent stochasticity and semantic drift typical in unconstrained generative augmentation.

As shown in Table 4, the proposed Propose–Select framework achieves the best overall data quality among all compared methods on the Oxford 102 Flowers dataset. Specifically, we analyze the augmentation performance from three dimensions:

- CLIP Sim: Propose–Select obtains the highest CLIP Sim score of 0.3325±0.0256, outperforming RandAugment (0.3113±0.0327), AugMix (0.3135±0.0337), TrivialAugmentWide (0.3094±0.0332), and DA-Fusion (0.3132±0.0279). This improvement demonstrates that the proposed cross-attention selector effectively exploits CLIP-based semantic priors to distinguish high-quality candidates from diffusion-generated samples with category deviation, such as unrealistic petal structures or inconsistent visual patterns.
- LPIPS: In terms of perceptual diversity, Propose–Select achieves an LPIPS score of 0.4839±0.0344, which is slightly higher than DA-Fusion (0.4813±0.0388) and substantially exceeds conventional augmentation methods such as RandAugment (0.4164±0.0791), AugMix (0.1504±0.0593), and TrivialAugmentWide (0.3369±0.1224). Unlike conventional methods that introduce diversity through fixed image transformations, Propose–Select expands the sample distribution through controllable generation, generating diverse flower appearances while preserving category-level semantic consistency.
- CLIP Feature Diversity: Our method achieves a CLIP Feature Diversity of 0.0808±0.0267, which demonstrates a stronger ability to maintain global feature richness compared to DA-Fusion (0.0721±0.0289) and AugMix (0.0630±0.0252). Although TrivialAugmentWide and RandAugment exhibit slightly higher CLIP Feature Diversity (0.0890 and 0.0864, respectively), this elevated diversity comes at the severe cost of semantic alignment (as evidenced by their significantly lower CLIP Sim scores). In contrast, Propose–Select achieves an optimal balance, effectively alleviating feature redundancy during sequential generation while strictly preserving crucial category-level semantics and structural fidelity.

#### 4.3.3. Training Dynamics and Generalization Analysis

**Figure 2:**
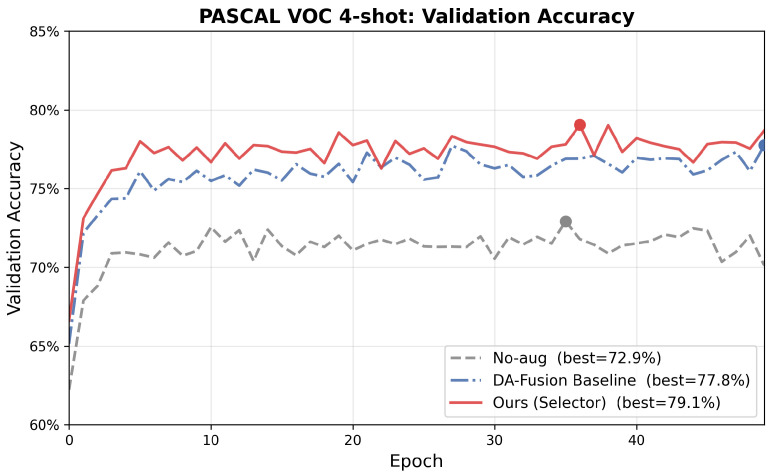
Validation Accuracy.

**Figure 3:**
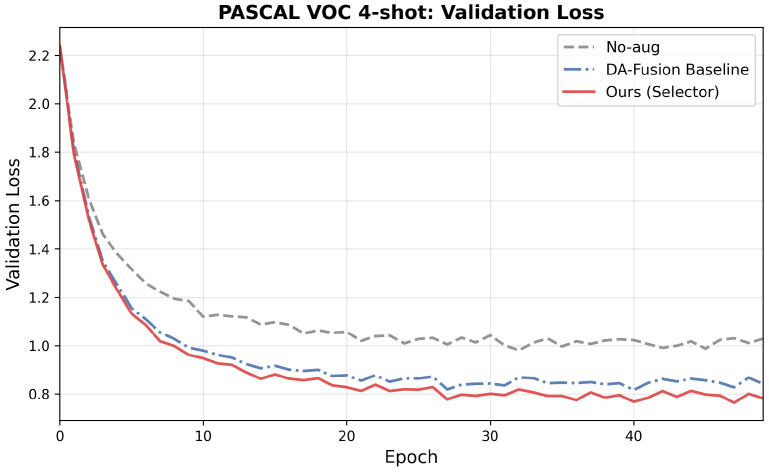
Validation Loss.

The validation accuracy and validation loss curves during training are illustrated in Figure 2 and Figure 3, respectively. As shown in Figure 2, under the No-aug setting, the accuracy quickly reaches a plateau at an early stage, indicating limited learning capacity due to the absence of data augmentation. The DA-Fusion baseline achieves noticeable improvements but exhibits certain fluctuations during training. In contrast, the proposed Propose–Select framework not only demonstrates a faster convergence rate but also maintains a consistently superior and stable performance throughout the entire training process.

The validation loss curves in Figure 3 further support these observations. The proposed method shows a more stable and smoother decrease in loss, ultimately converging to a lower value. This indicates that the filtered and high-quality augmented samples contribute to improved training stability and better generalization capability of the model.

**Figure 4:**
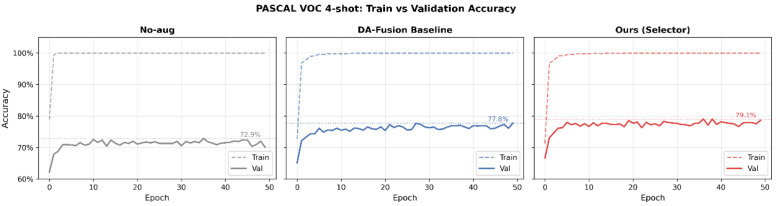
Train vs Validation Accuracy.

In addition, we compare the generalization gap between training and validation performance across the three methods, as shown in Figure 4. The No-aug baseline exhibits a clear overfitting behavior, where the training accuracy rapidly converges to 100% while the validation accuracy remains at only 72.9%. Although DA-Fusion improves validation performance to 77.8%, a noticeable discrepancy between training and validation accuracy still persists. In contrast, the proposed method not only boosts validation accuracy to 79.58%, but also effectively reduces the generalization gap, demonstrating stronger generalization capability.

### 4.4. Ablation Study and Parameter Analysis

**Figure 5:**
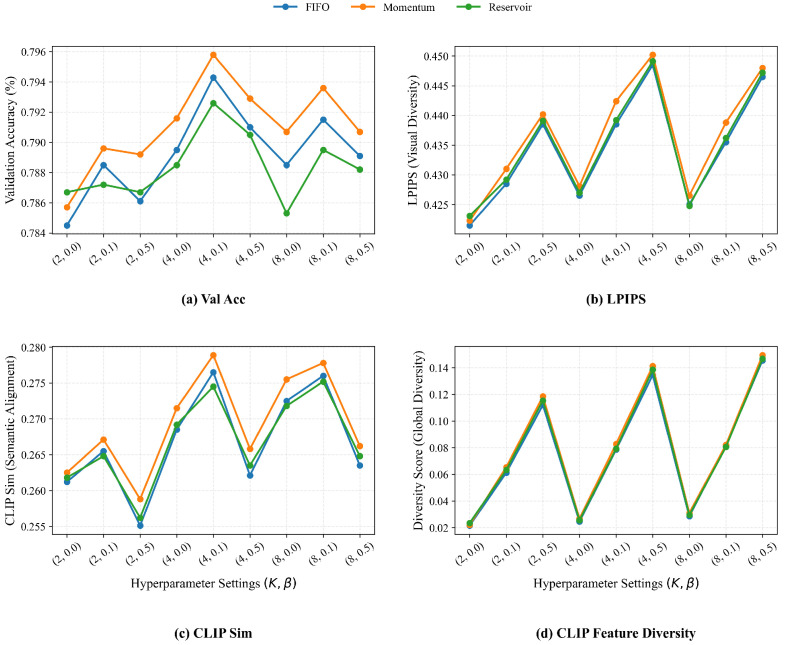
Performance comparison across different configurations.

To comprehensively evaluate the effectiveness and robustness of the proposed Propose–Select framework, joint experiments are conducted across multiple key dimensions on the PASCAL VOC 2012 dataset. The results are projected into a unified coordinate space for comparative analysis, as illustrated in Figure 5. The x-axis represents progressively varying (k,β) parameter configurations, while the y-axis illustrates the corresponding variations in evaluation metrics.

#### 4.4.1. Ablation on the Number of Candidates K

The number of candidates K determines the capacity of the selection pool and thus affects the likelihood of identifying high-quality images. When β=0.1 and the memory update strategy is set to Momentum, the multi-dimensional evaluation results under different values of K are reported in Table 5.

From Table 5, several observations can be drawn.

When K=2, the model achieves a validation accuracy of 78.96%, indicating that even a small candidate pool can provide improvements in the quality of augmented data. However, due to the limited number of candidate samples, further performance gains remain constrained.

When the candidate size increases to K=4, the model obtains the best overall performance across all metrics. Specifically, the validation accuracy improves to 79.58%, while LPIPS, CLIP similarity, and diversity score reach 0.4424, 0.2789, and 0.0828, respectively. This suggests that a moderate candidate pool size provides a better trade-off among semantic consistency, diversity, and global feature richness, enabling the selector to more effectively identify high-quality and diverse augmented samples.

When K=8, although CLIP similarity and diversity score remain relatively high, the validation accuracy slightly decreases to 79.36%. This indicates that an overly large candidate pool introduces increased semantic variability while also raising the likelihood of low-quality or noisy samples. As a result, the selection process becomes more challenging and computationally expensive, leading to diminishing performance gains.

#### 4.4.2. Diversity Penalty Weight β

The diversity penalty weight β plays a critical role in mitigating the tendency of diffusion models to repeatedly generate images with similar styles or poses. Table 6 reports the impact of different values of β on model performance.

When β=0, the model does not incorporate any diversity penalty mechanism. Although relatively high semantic consistency is maintained, achieving a CLIP similarity of 0.2715, the CLIP Feature Diversity is only 0.0268, indicating a high degree of feature redundancy among generated samples. Overall, the augmented samples tend to be homogeneous, and the model is prone to selecting instances that are highly similar to existing ones.

When β=0.1, the model achieves the best overall performance across all metrics. Specifically, the validation accuracy improves to 79.58%, CLIP similarity reaches its highest value of 0.2789, and the CLIP Feature Diversity increases to 0.0828.

When the penalty weight is further increased to β=0.5, both LPIPS and CLIP Feature Diversity rise to 0.4502 and 0.1412, respectively, indicating that the model generates more diverse visual variations. However, CLIP similarity decreases to 0.2658, and validation accuracy slightly drops to 79.29%. This suggests that overly strong diversity constraints may introduce excessive variation, causing partial semantic drift away from the target categories and ultimately degrading classification performance.

Overall, the experimental results demonstrate that the proposed diversity penalty mechanism effectively alleviates the homogenization problem in generated samples, introducing richer and more diverse visual variations while maintaining semantic consistency.

#### 4.4.3. Memory Bank Update Strategy (MEM)

The memory bank update mechanism directly determines how the model captures historical generation states, which is crucial for accurately computing the diversity penalty associated with β. The First-In-First-Out (FIFO) strategy maintains recent memory through a fixed-size sliding window. The reservoir sampling strategy introduces a long-range global perspective by randomly replacing stored samples, thereby improving coverage over the entire data distribution. The momentum strategy employs exponential moving average (EMA) to integrate features, providing each class with a smooth and noise-robust class prototype, which typically yields a higher performance upper bound.

As shown in Table 7, we extensively evaluated different memory bank update strategies. The FIFO (First-In, First-Out) strategy yields the lowest CLIP Feature Diversity (0.0785) among the three. This can be attributed to the inherent “recency bias” of the FIFO mechanism. Since it merely maintains a highly localized observation window of the most recently selected samples, it struggles to preserve the feature diversity representation once early diverse samples are flushed out. Conversely, the Momentum strategy accumulates a global moving average of historical features, maintaining a stable, long-term global receptive field. This prevents the memory bank from being overridden by transient generation homogeneity, thereby preserving a higher CLIP Feature Diversity and overall classification accuracy.

#### 4.4.4. Component Ablation of Composite Loss

To validate the necessity and mutual complementarity of the four regularization objectives in our composite loss function, we conducted a systematic component ablation study on the PASCAL VOC dataset. Consistent with our main evaluation protocol, all reported metrics in this ablation study represent the average results over five independent runs. As shown in Table 8, removing or reducing any single loss term results in noticeable performance degradation, confirming that existing single-objective screening methods are insufficient.

- Impact of Listwise Ranking Loss ($L_{rank}$): When $L_{rank}$ is entirely removed ($\lambda_{rank}=0$), the validation accuracy experiences the most severe drop (−1.50%, falling to 78.08%). This highlights that the relative quality ranking distilled from CLIP is the core driving force of the selection mechanism.
- Impact of Semantic Consistency Loss ($L_{sem}$): Removing $L_{sem}$ leads to a significant decrease in semantic alignment (CLIP Sim drops from 0.2789 to 0.2594) alongside a 0.94% drop in accuracy. This proves that without absolute semantic regularization, the selector is prone to selecting images that are visually appealing but semantically misaligned with the target class.
- Impact of Diversity Regularization Loss ($L_{div}$): When $L_{div}$ is deactivated, the feature diversity metric (CLIP Feature Diversity) drops notably from 0.0863 to 0.0828. This empirical evidence supports our claim that without an explicit memory-based diversity penalty, the generator tends to fall into mode collapse, producing highly homogeneous samples that limit the downstream classifier’s generalization.
- Impact of Permutation Consistency Loss ($L_{perm}$): The removal of $L_{perm}$ causes a substantial 1.16% decline in validation accuracy. This confirms that sequence dependency is a critical vulnerability in set-based evaluation. Enforcing permutation equivariance successfully stabilizes the selector training against arbitrary input orders.

In summary, the full model achieves the Pareto-optimal balance across classification accuracy (79.58%), perceptual diversity (0.4424), and semantic alignment (0.2789), demonstrating that the four loss components are indispensable and highly complementary.

### 4.5. Computational Efficiency Analysis

To verify the efficiency of our Propose–Select framework, we compare the computational overhead with the DA-Fusion baseline. Unlike end-to-end training methods, our framework adopts an offline selection paradigm.

- Selection Efficiency: The selection process is performed once offline. Our Cross-Attention Selector processes K=4 candidates in approximately 0.12 s per source image. Given that the diffusion generation itself typically takes 2.5 s per image, the selector adds less than 5% to the total offline augmentation time, which is negligible considering the significant quality improvements.
- Downstream Training Overhead: Our framework introduces zero additional overhead during downstream classifier training. Since selection occurs offline, the downstream model only trains on the pre-curated, high-quality subset. Consequently, the training time (s/epoch) is identical to training on a fixed-size dataset, validating our claim of no extra downstream training overhead.

Table 9 summarizes all major hyperparameters used in the proposed framework, including diffusion generation, selector architecture, loss coefficients, and training configurations, to facilitate reproducibility.

## 5. Conclusions

In this work, we have presented a novel two-stage Propose-and-Select framework to overcome the fundamental bottlenecks of unconstrained generative data augmentation in few-shot image classification. By transitioning from the conventional “generate-once, use-directly” paradigm to an offline filtration paradigm, this work successfully mitigates the long-standing challenges of uncontrollable sample quality, semantic drift, and mode collapse. The main contributions of this work are summarized as follows:

- Decoupled Candidate Selection Paradigm: Transforming unconstrained generation into a structured candidate-selection pipeline significantly elevates the reliability and quality bound of synthetic data. Our framework demonstrates robust performance gains across diverse domains, achieving a final accuracy of 79.58% on the natural PASCAL VOC benchmark and 80.74% on the fine-grained Oxford 102 Flowers dataset.
- A lightweight Cross-Attention Selector is developed. By leveraging the zero-shot prior knowledge of vision–language models such as CLIP, pseudo-labels are constructed without requiring additional human annotations. The selector integrates explicit quality signals, contextual cross-attention representations, and candidate-level interactions to achieve accurate quality ranking and candidate selection.
- A class-level Diversity Memory Bank is introduced to dynamically penalize redundant samples during sequential decision-making, thereby ensuring dataset-level diversity from a global perspective.
- A composite training objective comprising four complementary loss functions is formulated, including adaptive-temperature listwise ranking distillation, semantic consistency regularization, diversity regularization, and permutation consistency regularization. These objectives jointly facilitate stable, permutation-equivariant, and highly reliable selector training.

## Figures and Tables

**Table 1:** Comparison results of different methods on the PASCAL VOC 2012 Dataset.

Method	Avg. Best Val Acc (%)	Epoch	Final Val Acc (%)
No-aug	72.91±0.81	35	70.1
RandAugment	73.02±0.49	45	72.74
AugMix	73.91±0.52	45	73.08
TrivialAugmentWide	73.02±0.43	30	72.33
DA-Fusion	77.65±0.64	27	77.8
Propose–Select (Ours)	79.58±0.26	36	78.7

**Table 2:** Comparison results of data quality on the PASCAL VOC 2012 dataset.

Method	LPIPS	CLIP Sim	CLIP Feature Diversity
RandAugment	0.5969±0.0466	0.2651±0.0179	0.2435±0.0445
AugMix	0.4666±0.0412	0.2688±0.0177	0.2342±0.0460
TrivialAugmentWide	0.5797±0.0857	0.2653±0.0178	0.2520±0.0428
DA-Fusion	0.4102±0.0321	0.2455±0.0274	0.1856±0.0470
Propose–Select	0.4424±0.0286	0.2789±0.0216	0.0828±0.0245

**Table 3:** Comparison results of different methods on the Oxford 102 Flowers dataset.

Method	Avg. Best Val Acc (%)	Epoch	Final Val Acc (%)
No-aug	72.50±0.68	38	70.60
RandAugment	70.57±0.45	38	69.90
AugMix	69.34±0.62	34	68.65
TrivialAugmentWide	70.20±0.58	38	70.12
DA-Fusion	78.50±0.49	29	77.40
Propose–Select (Ours)	80.74±0.32	39	78.60

**Table 4:** Comparison results of data quality on the Oxford 102 Flowers dataset.

Method	LPIPS	CLIP Sim	CLIP Feature Diversity
RandAugment	0.4164±0.0791	0.3113±0.0327	0.0864±0.0292
AugMix	0.1504±0.0593	0.3135±0.0337	0.0630±0.0252
TrivialAugmentWide	0.3369±0.1224	0.3094±0.0332	0.0890±0.0337
DA-Fusion	0.4813±0.0388	0.3132±0.0279	0.0721±0.0289
Propose–Select	0.4839±0.0344	0.3325±0.0256	0.0808±0.0267

**Table 5:** Multi-dimensional Metrics under Different Values of K.

Method	K	β	MEM Strategy	Val Acc (%)	LPIPS	CLIP Sim	CLIP Feature Diversity
Propose–Select	2	0.1	Momentum	78.96±0.57	0.4310±0.0304	0.2671±0.0262	0.0654±0.0273
Propose–Select	4	0.1	Momentum	79.58±0.26	0.4424±0.0286	0.2789±0.0216	0.0828±0.0245
Propose–Select	8	0.1	Momentum	79.36±0.36	0.4388±0.0297	0.2778±0.0245	0.0821±0.0251

**Table 6:** Performance Comparison under Different Values of β.

Method	K	β	MEM Strategy	Val Acc (%)	LPIPS	CLIP Sim	CLIP Feature Diversity
Propose–Select	4	0.0	Momentum	79.16±0.43	0.4281±0.0314	0.2715±0.0272	0.0268±0.0269
Propose–Select	4	0.1	Momentum	79.58±0.26	0.4424±0.0286	0.2789±0.0216	0.0828±0.0245
Propose–Select	4	0.5	Momentum	79.29±0.31	0.4502±0.0292	0.2658±0.0238	0.1412±0.0258

**Table 7:** Performance Comparison under Different MEM Strategies.

Method	K	β	MEM Strategy	Val Acc (%)	LPIPS	CLIP Sim	CLIP Feature Diversity
Propose–Select	4	0.1	FIFO	79.43±0.52	0.4385±0.0304	0.2765±0.0257	0.0785±0.0252
Propose–Select	4	0.1	Momentum	79.58±0.26	0.4424±0.0286	0.2789±0.0216	0.0828±0.0245
Propose–Select	4	0.1	Reservoir	79.26±0.34	0.4392±0.0289	0.2745±0.0221	0.0792±0.0250

**Table 8:** Component Ablation of the Composite Loss Function (Average of 5 Runs).

Ablation Group	Setting	$\lambda_{\mathrm{rank}}$	$\lambda_{\mathrm{sem}}$	$\lambda_{\mathrm{div}}$	$\lambda_{\mathrm{perm}}$	Val Acc (%)	LPIPS (±σ)	CLIP Sim (±σ)	CLIP Feature Diversity (±σ)
(1) $L_{rank}$	Removed	0.00	0.10	0.10	0.050	78.08 (−1.50)	0.4224±0.0217	0.2689±0.0220	0.0808±0.0215
	Partial	0.50	0.10	0.10	0.050	78.71 (−0.87)	0.4341±0.0203	0.2635±0.0233	0.0842±0.0203
	Full	1.00	0.10	0.10	0.050	79.58 (0.00)	0.4424±0.0201	0.2789±0.0240	0.0863±0.0193
(2) $L_{sem}$	Removed	1.00	0.00	0.10	0.050	78.64 (−0.94)	0.4296±0.0195	0.2594±0.0186	0.0868±0.0203
	Partial	1.00	0.05	0.10	0.050	79.19 (−0.39)	0.4386±0.0200	0.2548±0.0206	0.0857±0.0200
	Full	1.00	0.10	0.10	0.050	79.58 (0.00)	0.4424±0.0201	0.2789±0.0240	0.0863±0.0193
(3) $L_{div}$	Removed	1.00	0.10	0.00	0.050	78.76 (−0.82)	0.4352±0.0200	0.2563±0.0230	0.0828±0.0201
	Partial	1.00	0.10	0.05	0.050	79.24 (−0.34)	0.4398±0.0214	0.2527±0.0218	0.0865±0.0217
	Full	1.00	0.10	0.10	0.050	79.58 (0.00)	0.4424±0.0201	0.2789±0.0240	0.0863±0.0193
(4) $L_{perm}$	Removed	1.00	0.10	0.10	0.000	78.42 (−1.16)	0.4364±0.0208	0.2535±0.0200	0.0868±0.0207
	Partial	1.00	0.10	0.10	0.025	79.00 (−0.58)	0.4382±0.0216	0.2525±0.0215	0.0863±0.0224
	Full	1.00	0.10	0.10	0.050	79.58 (0.00)	0.4424±0.0201	0.2789±0.0240	0.0863±0.0193
Full Model	Reference	1.00	0.10	0.10	0.050	79.58	0.4424±0.0201	0.2789±0.0240	0.0863±0.0193

**Table 9:** Systematic hyperparameter configurations and implementation details of the Propose–Select framework.

Category	Hyperparameter	Value	Description
Selector Architecture	Input Dimension ($d_{in}$)	512	CLIP ViT-B/32 embedding dimension
	Attention Heads (*H*)	8	Number of heads in Cross-Attention
	Selector Layers (*L*)	1	Number of stacked attention layers
	Dropout Rate	0.1	Dropout layer ratio for regularization
Classifier Optimization	Optimizer	SGD	Optimization algorithm for downstream model
	Learning Rate ($\eta_{cls}$)	0.01	Initial base learning rate
	Momentum	0.937	SGD momentum coefficient
	Weight Decay	0.0005	$L_{2}$ weight regularization factor
	Total Epochs	50	Downstream classifier training epochs
	LR Scheduler	Cosine	Cosine annealing with 10-epoch warm-up
Loss & Augmentation	Candidate Pool Capacity (*K*)	8	Number of proposed samples per step
	Diversity Weight (β)	0.05	Penalty factor for historical redundancy
	Min Temperature ($\tau_{\min}$)	0.003	Minimum temperature for listwise distillation
	Default Weights ($\lambda_{rank},\lambda_{sem},\lambda_{div},\lambda_{perm}$)	1.0, 0.1, 0.1, 0.05	Trade-off weights for composite objectives

## Data Availability

The code and pretrained model weights supporting the findings of this study are provided as Supplementary Material (Files S1 and S2). The original contributions presented in this study are included in the article. All technical inquiries regarding the implementation can be sent to the first author.

## Supplementary Materials

The following supporting information can be downloaded at: https://www.mdpi.com/article/10.3390/s1010000/s1.
